# Spatially-Controllable Hot Spots for Plasmon-Enhanced Second-Harmonic Generation in AgNP-ZnO Nanocavity Arrays

**DOI:** 10.3390/nano8121012

**Published:** 2018-12-05

**Authors:** Shaoxin Shen, Min Gao, Rongcheng Ban, Huiyu Chen, Xiangjie Wang, Lihua Qian, Jing Li, Zhilin Yang

**Affiliations:** 1 Department of Physics, Collaborative Innovation Center for Optoelectronic Semiconductors and Efficient Devices, Xiamen University, Xiamen 361005, China; ssx@stu.xmu.edu.cn (S.S.); mgao@stu.xmu.edu.cn (M.G.); bluesky530970142@gmail.com (R.B.); hychen@stu.xmu.edu.cn (H.C.); xjwang@stu.xmu.edu.cn (X.W.); 2 College of Information Science and Engineering, Fujian Provincial Key Laboratory of Light Propagation and Transformation, Huaqiao University, Xiamen 361021, China; 3 School of Physics, Huazhong University of Science and Technology, Wuhan 430074, China; lhqian@mail.hust.edu.cn; 4 Department of Physics, Pen-Tung Sah Micro-Nano Institute of Science and Technology, Xiamen University, Xiamen 361005, China

**Keywords:** plasmonics, second-harmonic generation, hybrid nanostructure, finite-difference time-domain

## Abstract

Plasmon-enhanced second-harmonic generation (PESHG) based on hybrid metal-dielectric nanostructures have extraordinary importance for developing efficient nanoscale nonlinear sources, which pave the way for new applications in photonic circuitry, quantum optics, and biosensors. However, the relatively high loss of excitation energies and the low spatial overlapping between the locally enhanced electromagnetic field and nonlinear materials still limit the promotion of nonlinear conversion performances in such hybrid systems. Here, we design and fabricate an array of silver nanoparticle-ZnO (AgNP-ZnO) nanocavities to serve as an efficient PESHG platform. The geometry of AgNP-ZnO nanocavity arrays provides a way to flexibly modulate hot spots in three-dimensional space, and to achieve a good mutual overlap of hot spots and ZnO material layers for realizing efficient SH photon generation originating from ZnO nanocavities. Compared to bare ZnO nanocavity arrays, the resulting hybrid AgNP-ZnO design of nanocavities reaches the maximum PESHG enhancement by a factor of approximately 31. Validated by simulations, we can further interpret the relative contribution of fundamental and harmonic modes to Ag-NP dependent PESHG performances, and reveal that the enhancement stems from the co-cooperation effect of plasmon-resonant enhancements both for fundamental and harmonic frequencies. Our findings offer a previously unreported method for designing efficient PESHG systems and pave a way for further understanding of a surface plasmon-coupled second-order emission mechanism for the enhancement of hybrid systems.

## 1. Introduction

Second-order responses, including second-harmonic generation (SHG), sum-frequency generation (SFG), and difference-frequency generation (DFG), give rise to wave-mixing effects that lead to frequency conversion [1]. The SHG, in which the fundamental frequency of the electromagnetic wave (ω) is converted to its second-harmonic (2ω), constitutes the most common phenomenon in nonlinear optics and often occurs in nonlinear crystals that are bulky and phase matched [1]. In past decades, optical devices based on several nonlinear crystals (such as β-BaB_2_O_4_) accomplish efficient SHG emissions. Recently, nanostructures with sub-wavelength geometries, such as BaTiO_3_ nanoparticles, ZnO, ZnTe, TiO_2_, CdS, KNbO_3_, and MoS_2_ nanowires, arrays of LiNbO_3_ nanocones, and lithium niobate (LN) nanorings, have been demonstrated to achieve high frequency-conversion efficiencies even if the phase matching condition is violated [2,3,4,5,6,7,8,9,10]. Unfortunately, the conversion efficiency of these nanostructures still remains low for their practical applications, implying that the high operation power is essential to generate sufficient signal emissions. The concentration of optical energy into nanometric volumes offers another way to dramatically enhance photon–electron interactions [11,12]. For this purpose, metal plasmonic metamaterials have been employed to provide locally enhanced electromagnetic fields [13,14,15,16,17]. Research regarding the excitation of localized surface plasmon resonances (LSPRs) and its influences on plasmon-enhanced SHG (PESHG) processes has become a significant topic because of its promising applications to nanoscale light sources [18,19,20]. Usually, the excitation of LSPRs results in hot spots featuring locally enhanced electromagnetic field in close vicinity to photon-excited nanostructures [21]. Engineering hybrid nanostructures that combine plasmonic nanostructures with materials with large second-order susceptibility gives an opportunity to realize the effective integration of strongly enhanced and independently tunable resonances and intense sources of SH polarization [18,22,23,24]. Particular interest lies in the fact that PESHG can be achieved due to the stimulated excitation of hot spots depending on the excitation and emission energies. However, evaluating the quantitative dependence between the spatial distribution of hot spots and the PESHG emission in these hybrid systems has rarely been reported.

In this work, we design and fabricate an array of silver nanoparticle-ZnO (AgNP-ZnO) nanocavities serving as an efficient PESHG platform. By controlling the spatial arrangement of AgNPs in three-dimensional space, the quantitative dependence between the spatial distribution of hot spots and the PESHG emission in the AgNP-ZnO nanocavity array has been demonstrated. Moreover, the near fields of Ag-NP dependent plasmonic resonances, both for fundamental and harmonic frequencies, are theoretically investigated by the 3D finite-difference time-domain (3D-FDTD) method, giving coherent experimental results for the PESHG signals.

## 2. Materials and Methods

### 2.1. Sample Preparation

**Figure 1:**
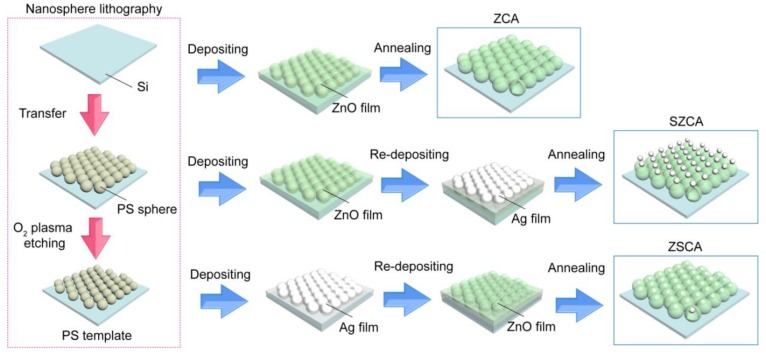
Fabrication procedures of arrays of ZnO nanocavities (ZCA), AgNP (on)-ZnO nanocavities (SZCA), and AgNP (in)-ZnO nanocavities (ZSCA).

Monolayered polystyrene (PS) sphere arrays based on nanosphere lithography were utilized as the supporting template (PS template) [25,26], and components of Ag and ZnO were prepared by radio-frequency magnetron sputtering deposition and subsequent thermal annealing (Figure 1) [27,28]. Mono-dispersed PS spheres with the diameter of 360 nm were synthesized using emulsion polymerization, so that their diameter deviations were less than 5% [29]. After that, the PS monolayer was patterned onto the clean Si substrate by convective assembly driven by solvent evaporation [30]. The size of PS spheres could be modulated via the O_2_ plasma etching with different durations to tune the separation of neighboring PS spheres. Then, thin Ag and ZnO films were sequentially deposited onto the PS template. After the depositions of Ag and ZnO films, these PS spheres were burnt off at 500 °C for 30 min in N_2_ ambient atmosphere. Three different kinds of arrays are fabricated, including arrays of ZnO nanocavities (ZCA), AgNP (on)-ZnO nanocavities (SZCA), and AgNP (in)-ZnO nanocavities (ZSCA), respectively. For ZCA, a 20-nm thick ZnO film was deposited onto the PS template, followed by thermal annealing. For SZCA, a 20-nm thick ZnO film and Ag films with the thicknesses of 10 nm, 20 nm, 30 nm, and 40 nm were deposited in sequence, and subsequent annealing induced the conversion of Ag film into AgNPs. For ZSCA, Ag films with four different thicknesses, 10 nm, 20 nm, 30 nm, and 40 nm, were initially deposited, followed by the deposition of a 20-nm thick ZnO film, and subsequent annealing induced the formation of AgNPs anchored onto the concave side of ZnO nanocavities. Morphologies and elemental compositions were characterized by scanning electron microscopy (SEM) images and energy dispersive X-ray spectra (EDS) on a Hitachi S-4800 field-emission scanning electron microscope (Hitachi, Tokyo, Japan). Note that the thermal treatment is helpful to improve the crystallinity of ZnO due to the Ostwald ripening effect in the annealing process, and thus all ZCAs indicate the preferential orientation along the wurtzite ⟨001⟩ direction [28].

### 2.2. SHG Measurement

**Figure 2:**
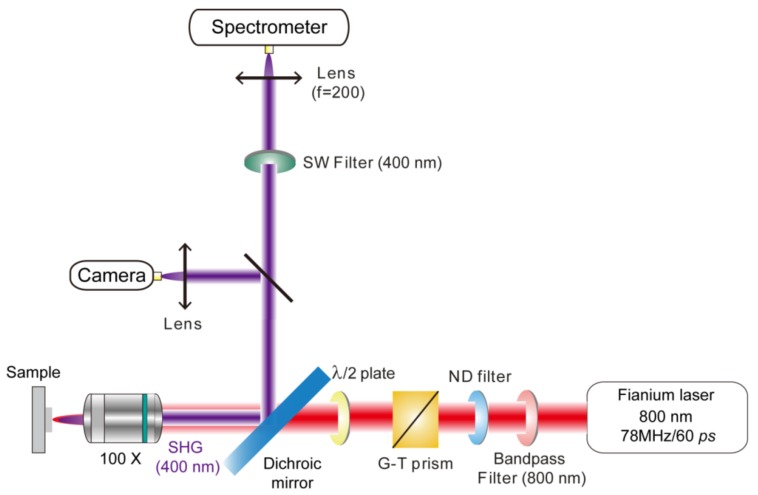
Experimental setups for the second-harmonic generation (SHG) measurement.

The reflection configuration (Figure 2) was constructed for the SHG measurement. A broadband Fianium laser equipped with an acousto-optic tunable filter and bandwidth-matched polarization optics (NKT photonics, pulse duration ~60 ps and repetition rate ~78 MHz) acted as the pumping source. A Glan-Taylor (G-T) prism and a half-wave plate were used to control the polarization of the incident laser. A bandpass filter at 800 nm was used to filter out the possible noise within the pumping source, and then the incident laser beam was focused by a 100× objective and illuminated on the sample in the perpendicular direction. Because the 1.5-μm diameter incident-beam spot is larger than the diameter of ZCA units, experimentally-observed SHG signals represent the average PESHG performance, enabling us to minimize the signals’ deviations from shape variations of individual sample units, and to rule out issues associated with the sample damage due to laser irradiation. The laser power can be controlled by continuously variable metallic neutral density filters ranging from 0.8 mW to 2.0 mW. The back-reflected SHG signal was collected by another lens (f = 200 mm) to focus the scattered beam. A short-pass filter at 400 nm (Semrock FF01-400/40) in front of the entrance slit of spectrograph (Horiba, iHR 550, Hamamatasu, Japan) was used to ensure that the transmittance (stopping power) in the fundamental wavelength was close to 0.0%, thus eliminating potential effects associated with stray light entering the monochromator in the SH spectra. The harmonic signals were then collected by a CCD detector (Horiba Symphony II) with an integral time of 30 s. Each SHG response has been maximized for comparison.

### 2.3. Computational Method

Near-field distributions within ZCA, SZCA, and ZSCA were simulated by commercial software, FDTD Solutions 8.7 (Lumerical Solutions, Vancouver, BC, Canada), which is based on the 3D-FDTD method [31,32]. The ZnO nanocavity with an inner diameter of 350 nm and 20 nm cavity thickness was hexagonally arranged. Here, we define SZCA or ZSCA deposited with a 10-nm, 20-nm, 30-nm, and 40-nm thickness Ag film as SZCA(10) or ZSCA(10), SZCA(20) or ZSCA(20), SZCA(30) or ZSCA(30), and SZCA(40) or ZSCA(40), respectively. For SZCA(10) or ZSCA(10), the elliptical AgNP had a longitudinal (x/y) axis of 130 nm and transverse (z) axis of 90 nm. For SZCA (20) or ZSCA(20), the elliptical AgNP had a longitudinal (x/y) axis of 200 nm and transverse (z) axis of 160 nm. For SZCA(30) or ZSCA(30), the elliptical AgNP had a longitudinal (x/y) axis of 270 nm and transverse (z) axis of 210 nm. Note that simulation parameters of AgNPs mentioned above were derived from SEM characterizations by means of the statistical analysis, and the direction of x-/y-/z-axis mentioned above is shown in relevant SEM images. A *p*-polarized plane wave is incident on the model at 90° and its electric-field amplitude was 1.0 V/m as the basis value for calculating the local electromagnetic field enhancement. The simulation time was 3000 fs to ensure calculation convergence. To obtain accurate results, the Yee cell size was set to be 1 × 1 × 1 nm^3^. To avoid the near-field interference between the incident and irradiated fields, perfectly matched layer boundary conditions were used for all simulations [33]. The number of perfectly matched layers depends on the mesh size in specific boundary conditions, and it was 12 for the 1-nm mesh size used in our case. Optical constants of Ag and ZnO were taken from the literature [34,35].

## 3. Results and Discussions

### 3.1. Fabrication and Characterization

#### 3.1.1. Morphology and Elemental Analysis

**Figure 3:**
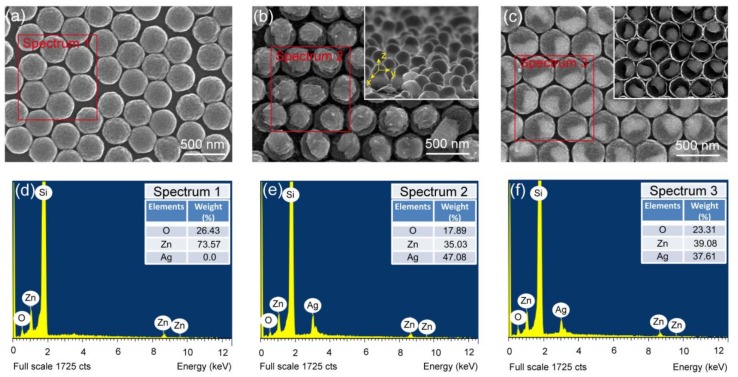
Top-view SEM images of (**a**) ZCA, (**b**) SZCA, and (**c**) ZSCA. The corresponding EDS spectra of (**d**) ZCA, (**e**) SZCA, and (**f**) ZSCA are also presented. The inset in (**b**) shows the side-view morphology of SZCA and the Cartesian coordinate system mentioned in the computational method. The inset in (**c**) refers to the inverted top-view morphology of ZSCA, indicating the trapping of AgNPs into ZnO nanocavities. The EDS spectra are collected from the regions marked with red boxes in (**a**–**c**), and the insets in (**d**–**f**) show the element weights determined from the corresponding EDS spectra. Note that in order to observe the interior structures of ZSCA, arrays on the Si substrate were lifted off by conductive tape and then inverted ZSCA were characterized.

The SEM images of ZCA, SZCA, and ZSCA made from PS templates with 360-nm period are presented in Figure 3. Because the size and the distribution of PS templates are modulated by O_2_ plasma etching for 10 s, the diameter of ZCA shows an average value of 350 nm (Figure 3a). Hexagonally arranged ZnO nanocavity arrays are uniform to some extent, guaranteeing the output of reproducible spectra shown in the following section. Figure 3b,c demonstrates the topographic characteristics of SZCA and ZSCA with a 20-nm-thickness Ag film, exhibiting different surface morphologies. The SZCA is produced with AgNPs capping on the convex surface of ZnO nanocavities (Figure 3b and inset), while AgNPs are on the concave surface of ZnO nanocavities in ZSCA (Figure 3c and inset). During the thermal annealing process, the Ag film tends to aggregate into many ellipsoids, whose dimensions are 200 nm, 200 nm, and 160 nm along x-, y-, and z-axes, respectively. The similar size of ZnO nanocavities can be visualized in all three cases. As shown in Figure 3b,c, the configuration of ZnO nanocavities can be served as a larger-area supporting template for AgNPs in SZCA and ZSCA [36,37]. The compositions of the three samples are characterized by EDS spectra (Figure 3d–f). Zn and O can be detected beside signals of the Si substrate. Ag can be distinctly observed in SZCA and ZSCA, indicating the generation of AgNPs in these hybrid systems (inset tables in Figure 3d–f). To distinguish different kinds of configurations more clearly, the Si signal can be used as a calibrated standard while calculating the concentration of the other elements. It is worth noting that the absence of C and H elements confirms that the PS spheres have been burnt off completely during the thermal annealing process.

### 3.2. Optical Spectroscopy and Simulations

#### 3.2.1. Geometry-Dependent PESHG Performances

**Figure 4:**
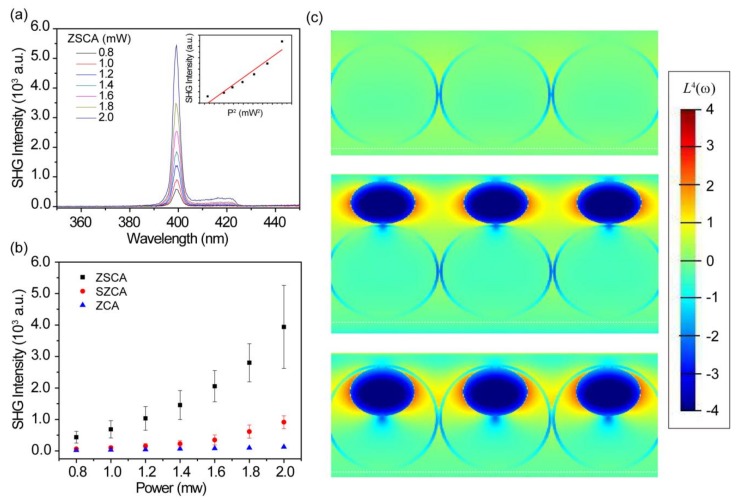
(**a**) Power-dependent plasmon-enhanced second-harmonic generation (PESHG): the emission intensity at second-harmonic (SH) wavelength increases quadratically with increased pumping power ranging from 0.8 mW to 2.0 mW. Inset: the measured PESHG intensity increases linearly with the square of pumping power (*P*^2^). (**b**) The comparison of power-dependent PESHG performances for ZSCA (black square dots), SZCA (red circle dots), and ZCA (blue triangle dots), respectively. Note that: the measured SHG intensity is a function of the excitation (average) power, which fits to a square dependence for all three samples. Error bars denote the deviation of the average SH signal intensity through multiple acquisitions from different spatial positions of the same sample. (**c**) Simulated near-field distributions of ZCA (**up**), SZCA (**middle**), and ZSCA (**bottom**) with a given excitation wavelength at 800 nm. All data are normalized.

For evaluating the nonlinear capability of AgNP-ZnO nanocavity arrays, we have obtained the power-dependent and geometry-dependent PESHG performances with a pumping wavelength at 800 nm. As expected, the signals collected from ZSCA increase quadratically with the increasing excitation power (Figure 4a) [38]. The inset of Figure 4a presents the measured SHG intensity as a function of the square of the pumping power *P*^2^. One can clearly see that the intensity increases linearly with *P*^2^, indicating the SHG response in our experiment [4]. The spectral position of the emission peak always appears at 400 nm, which is the half of the excitation wavelength [39]. The peak intensity increases as a function of the square of the pumping power *P*^2^ (inset of Figure 4a), further confirming the characteristics of SHG [4]. The experimental result of geometry-dependent PESHG performances demonstrates that the signal intensity of ZSCA exhibits the maximum value compared to SZCA and ZCA with a given excitation power ranging from 0.8 mW to 2.0 mW (Figure 4b). Because of the same ZnO crystalline phase in the sample preparation process, the SH polarization source originating from ZnO nanocavities in our experiments would possess similar nonlinear capability. To quantitatively estimate the PESHG ability of AgNP-ZnO nanocavity arrays, the SH enhancement factor ($\gamma_{EF}$) can be introduced by comparing measured PESHG intensities achieved on ZSCA, and ZCA [10]:

(1) $$\gamma_{EF}=\frac{I_{2\omega \;\mathrm{ZSCA}\;}}{I_{2\omega \left(\mathrm{ZCA}\right)}},$$

the $\gamma_{EF}$ yields a factor of approximately 31 at a given excitation power (*P* = 2.0 mW), revealing that compared to bare ZnO nanocavity arrays, the resulting hybrid AgNP-ZnO design of nanocavities reaches one-order enhancement of PESHG emission intensities. Moreover, in light of PESHG mechanisms, the PESHG enhancement factor (PESHG-EF) is defined as:

(2) $$M\left(\omega ,2\omega \right)={\left|L\left(2\omega \right)\right|}^{2}{\left|L\left(\omega \right)\right|}^{4},$$

where L(Ω), Ω=ω or 2ω refers to the local electromagnetic field enhancement through $L\left(\omega \right)=E_{\mathrm{loc}}\left(\omega \right)/E_{0}\left(\omega \right),\;\Omega =\omega \;\mathrm{or}\;2\omega$, where $E_{\mathrm{loc}}\left(\omega \right)$ and $E_{0}\left(\omega \right)$ are the local field amplitude and electric far-field amplitude at excitation and reemission frequencies, respectively. With Equation (2), one can estimate the magnitude of PESHG-EFs that originates from locally electromagnetic field enhancements, and analyze how the distribution of hot spots contributes to PESHG performances at either the fundamental frequency (excitation step) or the harmonic (reemission step) frequency [14,40,41,42,43].

To further understand the inherent physical mechanism, we conduct 3D-FDTD simulations to calculate the near-field distribution of ZCA, SZCA, and ZSCA at a given excitation wavelength (800 nm) (Figure 4c). As we can see, there is nearly no local-field enhancement around ZnO nanocavities, while a strong dipolar near field enhancement of AgNPs can be observed at an 800-nm excitation wavelength both for two asymmetric nanostructures (SZCA(20) and ZSCA(20)). For SZCA, strong near fields distribute on the region above the ZnO nanocavity array due to AgNPs situate on the convex surface of ZnO nanocavities. For ideally distributed AgNPs, there is no local-field enhancement in the bulk of the ZnO nanocavities. However, the inhomogeneous distribution of AgNPs in our experiments results in the partially spatial overlap between the near-field distribution of AgNPs and the bulk of ZnO nanocavities (Figure 3b), causing the slight increase of PESHG signal emissions above those of ZCA. For ZSCA, the AgNP is anchored at the concave surface of ZnO nanocavities. The dipolar local-field distribution extends into the region where the curved ZnO layer is coated; thereby, more spatial volume of ZnO nanocavities is located at the electromagnetic field with local enhancement. The difference of local electromagnetic field enhancements between SZCA and ZSCA is very small; therefore, we can attribute the observed PESHG signal to the larger bulk nonlinear susceptibility of ZnO nanocavities, and the significant increase of PESHG emissions in ZSCA stems from the effective spatial overlap between the dipolar local-field distribution of AgNPs and the ZnO nanocavity. Experimental results further show that the SHG emission originating from bare AgNPs on the Si substrate is negligible under our experimental conditions (data not shown here).

**Figure 5:**
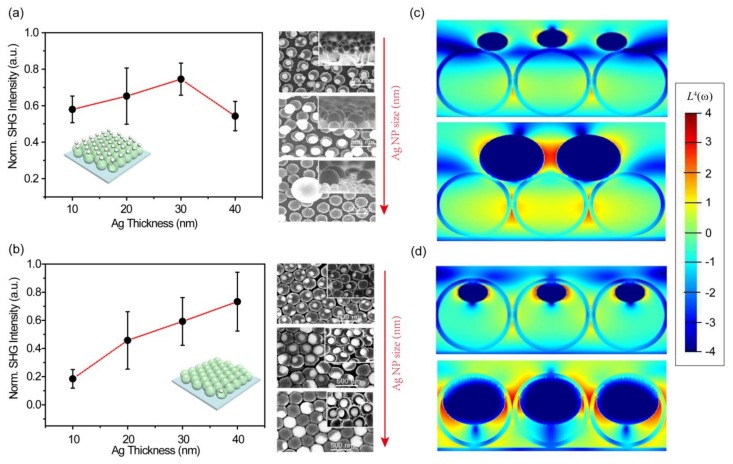
(**a**) Ag-NP dependent PESHG performances for SZCA(10), SZCA(20), SZCA(30), and SZCA(40) at a given power value (P = 1.4 mW). Insets: Ag-NP dependent SEM images of SZCA(10), SZCA(30), and SZCA(40), respectively. The red arrow refers to the trend of the coarsening of the AgNP size both in SZCA and ZSCA. The data in (**a**) are normalized by the maximum value of SZCA(30). (**b**) Ag-NP dependent PESHG performances for ZSCA(10), ZSCA(20), ZSCA(30), and ZSCA(40) at a given power value (P = 1.4 mW). Insets: Ag-NP dependent SEM images of ZSCA(10), ZSCA(30), and ZSCA(40), respectively. The data in (**b**) are normalized by the maximum value of ZSCA(40). Error bars in (**a**,**b**) denote the deviation of the average signal intensity through multiple acquisitions from three different spatial positions of the same sample. The red dashed lines are to guide the eyes. Simulated near-field distributions of (**c**) ZSCA(10) and ZSCA(30); (**d**) SZCA(10) and SZCA(30) were excited by an 800-nm pumping wavelength in logarithmic scale. All data are normalized.

To verify our assumption, we investigated Ag-NP dependent PESHG performances for SZCA and ZSCA, respectively (Figure 5). The SEM characterizations of Ag-NP dependent samples including SZCA(10) or ZSCA(10), SZCA(30) or ZSCA(30), and SZCA(40) or ZSCA(40) demonstrate that the size of AgNPs gradually increases, both in SZCA and ZSCA (insets of Figure 5a,b). It should be noted that AgNPs might finally aggregate into clusters in SZCA(40), resulting in the spatial absence of AgNPs on the convex surface of ZnO nanocavities. The PESHG intensities are recorded by manually choosing different sample areas to represent the deviation of the average PESHG performance. For SZCA, we collect peak values of PESHG intensities of SZCA(10), SZCA(20), SZCA(30), and SZCA(40), respectively (Figure 5a). A monotonic increment of PESHG intensities with the thickening of Ag films ranging from 10 nm to 30 nm can be observed with a given power (1.4 mW). A dramatic reduction can be obtained when we keep increasing the Ag-film thickness up to 40 nm. A similar trend has been shown with different pumping powers ranging from 0.8 mW to 1.8 mW (see Figure S1 in the supporting information (SI)). For ZSCA, Ag-NP dependent PESHG performances exhibit a monotonic increment in PESHG intensities with the thickening of deposited Ag films ranging from 10 nm to 40 nm (Figure 5b). In our experiments, the signal intensity of ZSCA(40) exhibits the maximum value among the samples, with a given excitation power ranging from 0.8 mW to 1.8 mW (see Figure S1 in SI). Due to the imperfection of the mono-dispersed PS template, there is still some large interparticle space, resulting in the heterogeneous distribution of deposited Ag and ZnO films. After the thermal treatment, the heterogeneous distribution of layered structures leads to the formed AgNPs being trapped into or onto ZnO nanocavities at certain vacant positions, resulting in the deviation of the average signal intensities through multiple acquisitions from different positions of the same sample. We further conduct 3D-FDTD simulations to calculate the near-field distribution of SZCA(10) and SZCA(30), ZSCA(10) and ZSCA(30) with a given excitation wavelength at 800 nm (Figure 5c,d), respectively. For SZCA, the coarsening of the AgNP size leads to the spatial redistribution of AgNPs on the convex surface of ZnO nanocavities, and the increasing PESHG intensity occurs when neighboring AgNPs are placed closely in the case of SZCA(30), as shown in Figure 5a. Meanwhile, because of the aggregation of AgNPs in SZCA(40), the spatial distribution of AgNPs on the convex surface of ZnO nanocavities become irregular, and the absence of partial AgNPs results in the poor spatial overlap between hot spots and the bulk of the ZnO nanocavities, inducing the decrement of PESHG signal emissions shown in Figure 5a. For ZSCA, the dipolar electromagnetic field distribution of AgNPs further extends into the bulk of neighboring ZnO nanocavities due to the enlargement of the AgNP size, and the high degree of spatial overlap between hot spots and the bulk of ZnO nanocavities contribute to the monotonic increment of PESHG emissions shown in Figure 5b. It is worth noting that, from the theoretical perspective, the influence of the period or nanocavity sizes on PESHG performances should be analogous to the discussion of Ag-NP dependent characterizations.

#### 3.2.2. Characteristics of Multimode Matching

**Figure 6:**
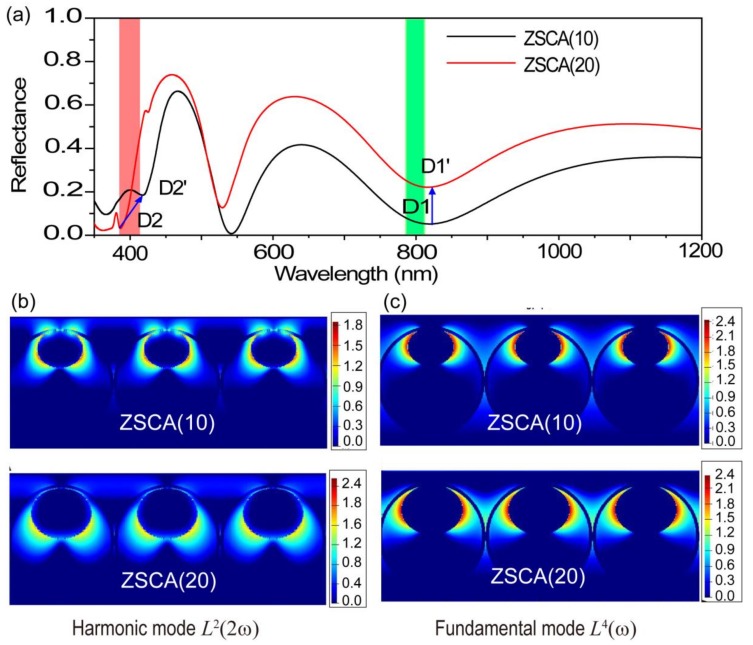
(**a**) Simulated reflectance spectra for ZSCA(10) and ZSCA(20). Simulated near-field distributions of Ag-NP dependent PESHG-EF: (**b**) harmonic mode (400 nm) for ZSCA(10) and ZSCA(20); (**c**) fundamental mode (800 nm) for ZSCA(10) and ZSCA(20) at the logarithmic scale.

For the characteristics of plasmonic modes to be elucidated, far-field reflectance spectra and near-field distributions in the visible and near-infrared regions for ZSCA(10) and ZSCA(20) can be calculated via conducting 3D-FDTD simulations (Figure 6). Based on the definition of PESHG-EF, we focus on two reflection dips: D1 and D2 (D1’ and D2’) for ZSCA near the 800- and 400-nm wavelength (marked by light green and red stripes), which cover fundamental and harmonic bands under our experimental conditions (Figure 6a). With the coarsening of the AgNP size, the red shift of dip D2 can be observed, while D1 remains constant (blue arrows in Figure 6a). As shown in Figure 6b,c, harmonic modes ($M\left(2\omega \right)={\left|L\left(2\omega \right)\right|}^{2}$) and fundamental modes ($M\left(\omega \right)={\left|L\left(\omega \right)\right|}^{4}$), both for ZSCA(10) and ZSCA(20), can be respectively assigned to the quadrupolar mode and the hybrid dipolar mode. The relevant near-field distribution of ZSCA(10) resembles that of ZSCA(20). However, the maximum value of the harmonic-mode amplitude of ZSCA(20) is almost one order of magnitude higher than that of ZSCA(10), while the maximum value of the fundamental-mode amplitudes in the two corresponding cases remains the same. Validated by our simulated results, we can reveal that the increasing PESHG intensity for ZSCA with the coarsening of the AgNP size stems from the local electromagnetic field enhancement at the harmonic frequency, and the co-cooperation effect of plasmon-resonant enhancements both for fundamental and harmonic frequencies makes the crucial contribution to the strong PESHG emission. To evaluate the harmonic field in plasmon-enhanced optical processes more accurately, the surface integral method, which takes into account nonlinear polarizations and boundary conditions at a nanoscale configuration surface, should be further considered [44].

## 4. Conclusions

In summary, we have demonstrated a two-dimensional AgNP-ZnO nanocavity array, which can be applied to reveal the quantitative dependence between the spatial distribution of hot spots and the PESHG emission in hybrid systems. The SH photons originating from ZnO nanocavities can be modulated by spatially-controllable hot spots in three-dimensional space, and efficiently generated due to a good mutual overlap of hot spots and ZnO material layers. Compared to bare ZnO nanocavity arrays, the resulting hybrid AgNP-ZnO design of nanocavities reaches the maximum PESHG enhancement by a factor of approximately 31 at a given average power. Simulated results confirm the relative contribution of fundamental and harmonic modes to Ag-NP dependent PESHG performances and verify that the cooperation effect of plasmon-resonant enhancements for both fundamental and harmonic frequencies makes the crucial contribution to strong PESHG emissions. This work provides a general rule for designing efficient platforms in PESHG and other nonlinear optics, which is one of the key functionalities for integrated photonic circuits.

## Supplementary Materials

The following are available online at http://www.mdpi.com/2079-4991/8/12/1012/s1, Figure S1: Ag-NP dependent PESHG performances for ZSCA and SZCA with different pumping powers.
