# Supplementary material for: Spatially-Controllable Hot Spots for Plasmon-Enhanced Second-Harmonic Generation in AgNP-ZnO Nanocavity Arrays
Source: Nanomaterials (Basel). 2018 Dec 5;8(12):1012. doi: 10.3390/nano8121012 (PMC6316233; doi:10.3390/nano8121012)
Supplement: Supplementary file 1 [file nanomaterials-08-01012-s001.pdf]

SUPPORTING INFORMATION

Article

Spatially-controllable hot spots for plasmon-enhanced second-harmonic generation in AgNP-ZnO nanocavity arrays

Shaoxin Shen [1,2,†], Min Gao [1,†], Rongcheng Ban [1], Huiyu Chen [1], Xiangjie Wang [1], Lihua Qian [4], Jing Li [3,*] and Zhilin Yang [1,*]

[1] Department of Physics, Collaborative Innovation Center for Optoelectronic Semiconductors and Efficient Devices, Xiamen University, Xiamen 361005, China; ssx@stu.xmu.edu.cn (S.S.); mgao@stu.xmu.edu.cn (M.G.); bluesky530970142@gmail.com (R.B.); hychen@stu.xmu.edu.cn (H.C.); xjwang@stu.xmu.edu.cn (X.W.)

[2] College of Information Science and Engineering, Fujian Provincial Key Laboratory of Light Propagation and Transformation, Huaqiao University, Xiamen 361021, China

[3] School of Physics, Huazhong University of Science and Technology, Wuhan 430074, China; lhqian@mail.hust.edu.cn

[4] Department of Physics, Pen-Tung Sah Micro-Nano Institute of Science and Technology, Xiamen University, Xiamen 361005, China

* Correspondence: lijing@xmu.edu.cn (J.L.); zlyang@xmu.edu.cn (Z.Y.); Tel.: +86-592-218-5962 (J.L.); +86-592-218-8510 (Z.Y.)

† These authors contributed equally to this work.

S1. Ag-NP dependent PESHG performances for ZSCA and SZCA with different pumping powers: The Ag-NP dependent PESHG performances for ZSCA and SZCA with different pumping powers ranging from 0.8 mW to 1.8 mW can be shown in Figure S2. We collect peak values of PESHG signals among these samples. For SZCA, a monotonic increasing trend of PESHG intensities with increasing the thickness of deposited Ag films ranging from 10 nm to 30 nm can be demonstrated with a given average power. A dramatic decrease of the PESHG intensity can be obtained when we keep increasing the thickness of the Ag film up to 40 nm. The similar trend can be obtained with different pumping powers ranging from 0.8 mW to 1.8 mW (Figure S2a). For ZSCA, an almost monotonic increasing trend of PESHG intensities with increasing the thickness of deposited Ag films ranging from 10 nm to 40 nm can be observed, and the maximum value of PESHG intensities occurs in the case of HZSCA(40). The same trend can be exhibited with different pumping powers ranging from 0.8 mW to 1.8 mW (Figure S2b).

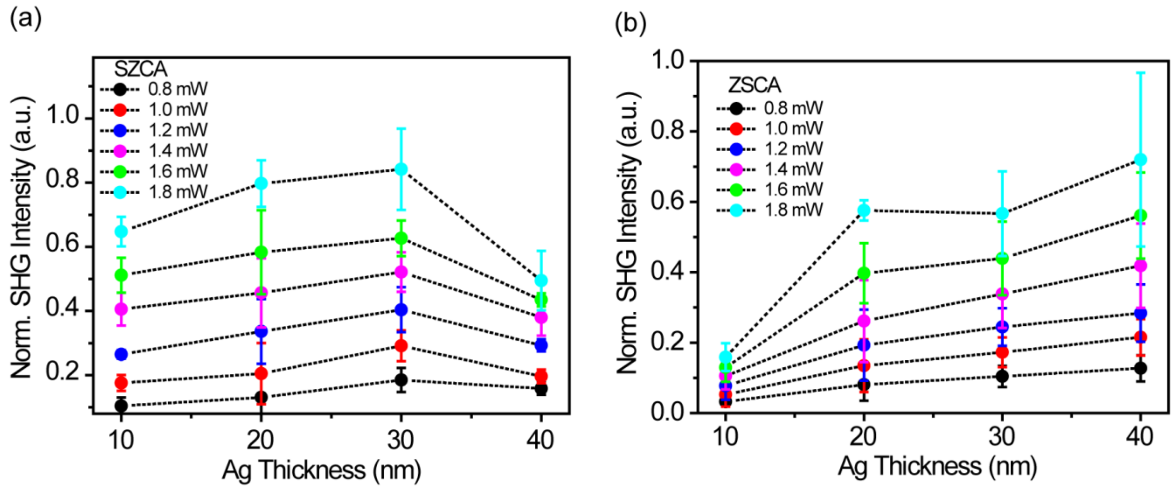

Figure S1. Ag-NP dependent PESHG for **(a)** SZCA and **(b)** ZSCA with different pumping powers: 0.8 mW (black dots), 1.0 mW (red dots), 1.2 mW (blue dots), 1.4 mW (magenta dots), 1.6 mW (green dots) and 1.8 mW (cyan dots), respectively. The data in **(a)** are normalized by the maximum value of SZCA(30) at the power of 1.8 mW, and the data in **(b)** are normalized by the maximum value of ZSCA(40) at the power of 1.8 mW. Error bars in **(a)** and **(b)** denote the deviation of the average signal intensity through multiple acquisitions from three different spatial positions of the same sample. The dotted lines are guided to the eyes.
